# “The angel of the house” in the realm of ART: feminist approach to oocyte and spare embryo donation for research

**DOI:** 10.1007/s11019-013-9513-1

**Published:** 2013-09-15

**Authors:** Anna Alichniewicz, Monika Michalowska

**Affiliations:** 1 Uniwersytet Medyczny w Lodzi, Lodz, Poland; 2 al. Kosciuszki 55 m.10, 90-514 Lodz, Poland; 3 ul. Piaseczna 5 m 17, 93-015 Lodz, Poland

**Keywords:** Gender stereotype, Exploitation, Oocyte and spare embryo donation for research, Autonomy and internalized oppression, Informed consent

## Abstract

The spectacular progress in assisted reproduction technology that has been witnessed for the past thirty years resulted in emerging new ethical dilemmas as well as the revision of some perennial ones. The paper aims at a feminist approach to oocyte and spare embryo donation for research. First, referring to different concepts of autonomy and informed consent, we discuss whether the decision to donate oocyte/embryo can truly be an autonomous choice of a female patient. Secondly, we argue the commonly adopted language of gift is misleading and that calling for altruism could put female patients at risk of exploitation. Finally, we point out that the presence of gender stereotypes in the procreative area casts doubt whether even a more robust notion of informed consent manages to overcome this risk.

For the past thirty years we have been witnessing a spectacular progress in assisted reproduction technology (ART). It has been generally argued that women are greatly benefited by ART, because it has enhanced their procreative opportunities. The progress in ART has been accompanied by a hot debate concerning its perspectives and moral controversies. Nevertheless, even if in the last years there have emerged several works whose authors addressed the possible threats ART poses to women (Carroll and Waldby 2012; Cohen 2007; Jayaprakasan et al. 2007; Nuffield Council on Bioethics 2011; Waldby 2008; Widdows 2010), these issues still have gained relatively lesser interest than other aspects. In our paper we are having a closer look at the problem of the forms of possible coercion women undergoing ART may face. We are especially interested in the pressure that could be placed on women to donate their oocyte or fresh embryo for research in the context of an IVF procedure. As far as ovum donation is concerned, women are often asked to donate it before their IVF cycle is completed because as stem-cell scientists admit: “we know we need to get the eggs immediately—we have to start the procedure within two hours after the eggs are retrieved” (Braun and Schultz 2012, p. 144). Being aware of the fact that oocyte and embryo donation is differently regulated by the national laws (for instance the US vs. Spanish systems) and the fact that in some countries this procedure is not allowed at all (e.g. Germany or Austria) we would like to provide a general view on some possible threats it posses to women regardless of the particular legal setting.

In the majority of bioethical companions the beneficial outcome of ART for patients is generally taken for granted with only a very superficial awareness of the risks they create for women in various aspects of their lives (e. g. Fulford et al. 2002). The analyses in this field have been mostly focused on the problems concerning the moral status of embryo, commercialization of oocyte market, reproductive labour, economic situation of women donating their eggs a well as a close institutional and personal integration of IVF and stem cell research fields (Braun and Schultz 2012; Dickenson 2006; Goold and Savulescu 2009; Haimes and Luce 2006; Haimes et al. 2008; Haimes and Taylor 2009; Schubert 2013; Waldby 2008). The ethical issues regarding somatic cell nuclear transfer (SCNT) and the moral status of the so-called research embryo have also gained some interest (Agar 2007; Devolder 2005; Green 2002; Holm 2002). Although some forms of possible exploitation and specifically coercion affecting women undergoing in vitro fertilization (IVF) have already been recognized and discussed (Donchin 1996; Purdy 1996) the majority of authors have pointed out only the fact that women’s decision to undergo IVF may not necessarily reflect their genuine needs. Apart from some notable exceptions (Carroll and Waldby 2012; Haimes et al. 2012; Scully et al. 2012; Waldby 2008), there has been almost no debate concerning detrimental outcomes resulting from IVF procedure offshoot that is oocyte and spare embryo donation for stem cell research. As Donna Dickenson notes, women often “become invisible in these procedures” (Dickenson 2003, p. 143). A constant call to investigate these problems coming from for instance bioethical blogs (Albany Medical College. Bioethics Today. 2013) indicates that the problem of the possible adverse effects for women consenting to donate their oocyte or spare embryo has not been given an adequate coverage so far. Moreover, it seems interesting to find that even if potential health, financial and emotional risks caused by egg and embryo donation are recognized, it is, nevertheless, claimed that properly obtained informed consent provides a guarantee that the patient’s autonomy is sufficiently secured (Haimes et al. 2013, p. 288; ASRM 2004, 2008).

It should be noted that in the process of gamete donation both women and men can be coerced or exploited. For instance, if male gametes were used against the donor’s will, his rights would be violated in the same way as the autonomy of a woman is disrespected when she is deprived of her right to consent or refuse to gamete donation. In the case of women, however, several additional factors contributing to the higher level of potential exploitation should be recognized. First, because female gametes constitute a scarce medical resource and therefore their market as well as research value is significantly high, some pressure to donate them could be imposed on potential donors. Secondly, there are significant differences between procedures of male and female gamete retrieval. In comparison to sperm retrieval, the medical procedure of oocyte harvesting creates a much greater health risk, let alone that it is also extremely burdensome. It has been already acknowledged that IVF procedures involving ovarian stimulation and medical procedure of retrieving oocytes pose serious health risks to women. One of the best-known disadvantageous outcomes is ovarian hyperstimulation syndrome (OHSS) that provokes both short- and long-term health disorders ranging from mild symptoms, like nausea or abdominal distension to life-threatening problems, like renal failure, or—as some findings recently suggested—ovarian cancer (Haimes et al. 2013, p. 286; McLeod and Baylis 2007, p. 468). However, the question is arising whether a woman in fact acts against her interests or exposes herself to a greater risk if she consents to donate spare oocytes or spare embryos for research. Let’s suppose that instead of deciding to have her spare oocyte or embryo frozen for possible later use, she has donated them. So, if the IVF appears unsuccessful, she will have to undergo the entire procedure again. Therefore, it can be stated that spare gamete or embryo donation is against her interest, because health risks become greater. It should be added that increased number of oocytes retrieval procedures means also greater financial and psychological burdens on IVF patients. As far as frozen embryo donation is concerned, it has been generally accepted that in the majority of cases the decision to donate it does not affect well-being of IVF patients. It could be, nevertheless, noted that also such a decision can appear against IVF patient’s interests in case she changes her mind and decides to have more progeny. With regard to that point Dickenson has observed that women’s decision of donation may not be autonomous because of two main reasons: (1) the recognition of women’s role in egg or embryo donation is clouded by gender stereotypes; (2) the risks are not sufficiently spelled out (Dickenson 2003, pp. 139–143). We will return later to the problem of gender stereotypes. Now we are going to sketch out the general background of the notion of autonomous decisions in gamete donation.

Ambiguous as it is, the notion of autonomy has become one of the fundamental concepts of bioethics. In his book on autonomy, Gerald Dworkin indicated that there are many understandings (and maybe misunderstandings) of this notion and many words can serve as synonyms, e.g. liberty, independence, freedom from coercion, to name just a few most typically used. The idea of autonomy has its proper place in the philosophical tradition, although—paradoxically—one of the two most often claimed authors, that is John Stuart Mill, hardly ever used the word ‘autonomy’. Nevertheless, it is him as well as Immanuel Kant, who are typically referred to as the ‘fathers’ of the idea. Of course, their accounts of autonomy differ profoundly, with Mill pointing mainly to liberty as the right to shape one’s life by his/her own independent choices and Kant focusing on his idiosyncratic notion of autonomy of practical reason. It seems that bioethics, at least initially, has been grounded first and foremost on Millan tradition.

It does not mean, however, that bioethicists have unanimously agreed on the theoretical background of the concept of autonomy. In her book *Autonomy and Trust in Bioethics*, Onora O’Neill argues that it is neither Kantian nor Millan account the bioethical use of the notion of autonomy is based upon, but mainly the one provided by twentieth psychology of character and moral development (O’Neill 2002, p. 23). She claims so because in bioethics the prevailing meaning of autonomy is that of independence. Thus, the medico-moral principle of respect for patient’s autonomy understood as patient’s independence is the one the rule of informed consent has stemmed from. As Neill Manson and Onora O’Neill put it: “informed consent procedures protect individual choice, and with it individual independence, hence individual autonomy” (Manson and O’Neill 2007, pp. 18–19).

Also, Ruth Faden and Tom Beauchamp say: “informed consent is rooted in concerns about protecting and enabling autonomous or self-determining choice by patients and subjects” (Faden and Beauchamp 1986, p. 235). Furthermore, two distinctive facets of this meaning of autonomy should be recognized, namely autonomy of person and autonomy of action, and it is the latter that has been declared by them to be the conceptual basis of the notion of informed consent. Faden and Beauchamp state that autonomy of person could be defined as having capacities to act consistently, independently, self-directedly and resistantly to any control by authorities (Faden and Beauchamp 1986, p. 236). They stress that it is the distinction between autonomous person and autonomous action that has been generally overlooked in bioethical literature, and indicate that even an autonomous agent may not act autonomously unless some specific conditions are met. They point out that “the capacity to act autonomously is distinct from *acting* autonomously, and possession of the capacity is no guarantee that an autonomous choice has been or will be made” (Faden and Beauchamp 1986, p. 237). In their view, there are three necessary conditions for an action to be considered autonomous, namely the agent must act intentionally, with understanding and without controlling influences (Faden and Beauchamp 1986, p. 238). Given that the goal of informed consent “is to enable patients and subjects (…) to make substantially autonomous choices about whether to authorize a medical intervention or research involvement” (Faden and Beauchamp 1986, p. 237), it can be admitted that informed consent meant as the operational rule of the principle of autonomy apparently matches that interpretation.

It has been often criticized that Faden and Beauchamp’s notion of informed consent is relatively narrow, and as such, it seems insufficient to guard the patient’s autonomy satisfactorily in medical setting. Focusing on the autonomy of action rather than that of person, they in fact have isolated decisions from the deciding person. Although it has been presented as the general concept of autonomous choice, in practice it has been reduced to a negative one, that is, to the right to refuse a proposed treatment. To overcome the weaknesses of this approach, it could be therefore claimed that it ought to be rather autonomy of person than autonomy of action upon which the rule of informed consent should be based. Such a concept of autonomy has been proposed by Dworkin in his *The Theory and Practice of Autonomy*. Developing Harry Frankfurt’s concept of the structure of the will (Frankfurt 1971), he says that “autonomy is conceived of as a second-order capacity of persons to reflect critically upon their first-order preferences, desires, wishes, and so forth and the capacity to accept or attempt to change these in light of higher-order preferences and values. By exercising such a capacity, persons define their nature, give meaning and coherence to their lives, and take responsibility for the kind of person they are” (Dworkin 1997, p. 20). In Dworkin’s view an autonomous agent is characterized by the capacity of self-reflection that allows him/her to give meaning to his/her life plans and actions and to shape his/her life in accordance with accepted moral values and principles. In that way he/she can be the true author of the narrative of his/her life.

There is, however, a more fundamental disagreement concerning the theory of autonomy. As Sonya Charles points out, Dworkin’s view on autonomy presents a classic example of a procedural concept of autonomy. Taking a feminist approach in her paper *How Should Feminists Autonomy Theorists Respond to the Problem of Internalized Oppression?*, she analyzes procedural and substantive concepts of autonomy. As she observes: “Procedural theorists claim that autonomy has to do with *how* a decision is made, not *what* decision is made”, that means that they “emphasize critical reflection, authenticity, and content neutrality” (Charles 2010, p. 411). According to Charles, the core idea of the procedural theory of autonomy is that it is the accordance with an established procedure that makes a given decision autonomous. A different approach is adopted in substantive theories where some “additional criteria such as requiring sufficient self-respect or self-worth or requiring specific content of beliefs or preferences” are included (Charles 2010, p. 409). Moreover, she points out that in the procedural theories exclusively subjective or ‘internal’ point of view is taken into account, whereas in the substantive ones “nonsubjective criteria or certain “external” value judgments” are involved (Charles 2010, p. 409).

What seems especially important for the problem we are dealing with is her opinion that the procedural approach to autonomy overlooks the problem of internalized oppression, that is, the fact that some forms of socialization are pernicious. As Charles puts it “Internalized oppression is internalized norms that lead a person to participate in perpetuating her own oppression” (Charles 2010, p. 410, n. 6). Thus, the procedural approach adopting merely subjective or internal perspective is seen not to be an adequate angle from which you can decide whether some decisions made by women are as autonomous.

The problem of a relationship between internal norms and social ideals is very complex and should not be reduced to a straightforward causal chain, nevertheless it has been generally acknowledged that our attitudes and choices are to much extent shaped by the process of socialization. It has been recognized that people’s behaviour and self-awareness are highly influenced by stereotypes, fixed opinions, prejudices and biases about themselves, in other words by popular ‘theories’ people encountered in the socialization process (Aronson 2011). In that way some leading stereotypes could become real in their effects.

Thus, the question arises which of these stereotypes could be harmful. To address the question, feminist authors argue the nonsubjective criteria must be employed. It deemed necessary to detect internalized oppressive norms resulting in nonautonomous preferences (Stoljar 2000; Charles 2010). Charles holds that if a decision is implied by false beliefs, it should not be treated as autonomous (Charles 2010, p. 413). However, to recognize the falsehood of a belief we need to step out from the internal perspective and take some distanced external one.

We argue that a generally accepted opinion that women are ‘by nature’ compassionate and caring is one of these false beliefs. It can be traced to the psychological concepts of Jean Piaget, Lawrence Kohlberg and Carole Gilligan. In the classical works of Kohlberg a six-stage scheme of moral development was presented (Kohlberg 1981). The final stage of moral development is that of universal ethical principles regarded as more fundamental than any institutional norms and legal acts could ever be. At this level a rational agent identifies himself with the set of norms and values and does not need any externally imposed obligation to respect them. In his theory, Kohlberg correlates epistemological and psychological development with moral progress that starts with perception of others from egoistic point of view and proceeds to rational, logically-based recognition of justice (Kohlberg 1981, p. 180). Gilligan criticized Kohlberg’s account as almost totally excluding women. They become silent and invisible and, moreover, judged according to the criteria based upon the empirical study carried out on boys and men. In her well-known work *In a Different Voice* Gilligan observed that performing moral judgment girls/women try to take into consideration the entire network of relationships they are involved in. Therefore, when evaluated by Kohlberg’s criteria they must be regarded as permanently unable to challenge authorities and reach the level of abstractive and logical moral reasoning. If judged according to Kohlberg’s criteria, they rarely, if ever, manage to achieve the post-conventional level, let alone the final stage.

Regardless of the argument that can be raised against it, Kohlberg’s study has become the general pattern of human moral development. When in her works Gilligan brought “into the masculine citadel of justice the feminine plea for mercy” (Gilligan 2003, p. 105), she presented the feminine moral development against the background constituted by Kohlberg’s study. Analyzing the responses to different moral dilemmas, Gilligan draws a conclusion that while for men responsibility is identified negatively as the limitation of their possibilities, women see it positively as a response to others and as an act of care. In Gilligan’s comparison of masculine and feminine morality we always encounter the tension between ‘morality of rights’ supporting individual claims and ‘morality of responsibility’ giving the priority to relationships. Moreover, we can notice that the majority of women Gilligan talked to appeared to be quite obsessed by fear of being selfish, because in their understanding selfishness remained in the strict opposition to responsibility. At some stage of moral development their notion of unselfishness becomes so demanding that in their view anything less than self-sacrifice seems selfish. In Gilligan’s opinion, it is only at the higher stages of feminine moral development when responsibility could be separated from self-sacrifice. She concludes that there are two different models of moral maturity resulted from two different developmental processes with feminine idea of maturity noticeably shifted from the one depicted in Kohlberg’s account. Comparing masculine and feminine notions of maturity, we find the former focusing primarily on a reflective understanding of human rights, and the latter emphasizing “the world of relationships”, Gilligan says (Gilligan 2003, p. 167).

Both Kohlberg’s and Gilligan’s studies have been criticized and further research has not found any significant differences between genders in their patterns of moral development (Mullet 1988; Bartky 1990). In our view, the evaluation of Gilligan’s findings first of all gives rise to a fundamental question whether it is really the case that the phenomena she has described are a cause rather than an effect. It seems that Gilligan has ignored the tremendous impact of gender stereotype ascribing altruism and unselfishness to ‘feminine nature’. Founding her model of feminine moral development on research carried out in the 70s of the twentieth century, she made her inquiries into moral development on the group of women raised on the cultural discourse that was ironically summarized by Virginia Woolf in her *Killing the Angel in the House*: “She was intensely sympathetic. She was immensely charming. She was utterly unselfish. She excelled in the difficult arts of family life. She sacrificed herself daily. If there was chicken, she took the leg; if there was a draught she sat in it—in short she was so constituted that she never had a mind or a wish of her own, but preferred to sympathize always with the mind and wishes of others” (Woolf 1995, p. 3).

Erik Malmqvist and Kristin Zeiler took a further step in their view on the process of socialization resulting in internalized norms. They argue that the problem lies not in the fact that the agent fails to recognize the oppressive norms she acts upon as false, but that she is unable to see them at all, that is, to recognize them as the norms she is motivated by. (Malmqvist and Zeiler 2010, p. 145). Taking a phenomenological approach, Malmqvist and Zeiler base their account of the problem of autonomy on Merleau-Ponty’s theory of the lived body, and state: “that many pervasive cultural norms are habitually incorporated” (Malmqvist and Zeiler 2010, p. 144) and as such elusive. These norms work on the taken-for-granted level and do not become the object of reflective analysis. Observing that these norms “evade the sort of self-reflective scrutiny of our motivational structure that is crucial to autonomous agency” (Malmqvist and Zeiler 2010, p. 144), Malmqvist and Zeiler admit, however, that even deeply incorporated social norms should not be regarded as totally determining. They draw the converse process of excorporation in which the concealed oppressiveness are to be revealed and reflectively pondered (Malmqvist and Zeiler 2010, pp. 145–150), and argue that excorporation starts “a process of *changing* pervasive norms, on an individual and societal level” (Malmqvist and Zeiler 2010, p. 149). However, it seems that the process does not go on easily and it encounters many obstacles.

Although the latest research shows that there have actually been some striking changes in social roles and gender images caused by economic and social-cultural transformations (Giddens 2009, 2012), there can be observed that traditional gender images are still visible and highly influential (Stivers 2002; Solinger 1998; Kukla 2008). It can be argued the models of moral development depicted in Kohlberg’s and Gilligan’s schemes have become ‘real in their effects’, since—despite their flaws and inadequacy—they continue to govern boys’ and girls’ upbringing, respectively. The gender models of upbringing remain overwhelming, because they support traditional essentialistic views on ‘masculine and feminine nature’, with emphasis on independence and self-focusing of the former and relationship involvement as well as self-sacrifice of the latter. And vice versa: the differences in moral and social upbringing petrify traditional feminine and masculine attributes, women as sensitive, emphatic and eager to nurture others’ needs and men as rather self-centered and inclined to tend to their own self-development. All that contributes to the fact that gender stereotypes die hard. The fact that they become recognized or ‘excorporated’ does not automatically imply that they are going to be refuted. Paradoxically, they happen to be even re-evaluated as the source of ‘girl power’. Announcing the commencement of a new matriarchy era, in *The End of Men and the Rise of Women* Hanna Rosin recognizes the traditional set of feminine attributes, like empathy, patience, flexibility and communal problem solving as the main factor contributing to their alleged present dominance (Rosin 2012).

It can be presumed that as choice-makers, female patients could be put in a difficult position by their ‘imprinted altruism’. As Dickenson has noticed, their situation is made even more problematic by ‘the language of gift’ that is commonly employed in the guidelines for oocyte donation and other ART procedures (Dickenson 2003, p. 143). Also, Theresa Glennon points out that “egg donation programmes emphasize altruism (…) Their rhetoric focuses on egg donation as a ‘gift’ exchange” (Glennon 2012, p. 101). The idea of gift is prevalent in the entire ‘reproductive market’ and it is most strikingly reflected in ART online advertisements always calling for altruistic help: “Give ‘the gift of heart’ to a person in need” (Invicta); “Welcome to gift ov life—setting egg donation standards” (Gift Ov Life); “We recognize that your decision to be an egg donor is a tremendous gift!” (Georgia Reproductive Specialists).

It seems that not only ART professionals are of the opinion that such advertisements are persuasive because this gender stereotype has influenced the laypeople’s views. Even more startling is the fact that it has its impact on the oocyte donation ethical guidelines which, as Dickenson notes, “uniformly employ the ‘language of gift’” (Dickenson 2003, p. 143). What is even more conspicuous, that belief is also shared by some professionals involved in philosophical and ethical disputes. For instance, it could seem that Cynthia B. Cohen in her book *Renewing the Stuff of Life* does recognize disadvantageous outcomes faced by women undergoing IVF. She points out that it is not only the question of medical risks, but in the case of women asked to donate their gametes for research it is also a question of possible coercion. Nevertheless, she still presupposes that the value of altruism is rewarding for women, saying: “Although women who provide eggs for research receive the altruistic satisfaction of knowing that they might assist others to overcome serious disease, they gain such satisfaction only at some risks to themselves” (Cohen 2007, p. 33).

In that way women become unwitting ‘victims’ of the set of moral virtues typically ascribed to them. Given how demanding the moral ideal set by these features is, it can be argued that in some decisional contexts this model could make women more susceptible to exploitation. Such a risk of exploitation can emerge in the procedure of gamete donation where a female patient has to make a choice between consenting to donate gametes for research, which is perceived as altruistic and therefore morally valued, or refusing to donate them, which is seen as incompatible with feminine ‘caring and empathic’ moral ideal and therefore morally flawed. Thus, the choice the patient has to make is either to act in her own interest and fail to match the moral ideal or to give priority to the needs of others and come up to the supererogative moral requirements. Ian Wilmut’s opinion seems to leave no doubts which decision women are expected to make: “I have never doubted that women would donate if they thought we were helping people to have treatment. Our hope and belief is that women who have seen the devastating effect of this disease will be prepared to make such a donation” (Sample and MacLeod 2005).

In our opinion, even the most ‘robust’ guidelines for informed consent do not solve the problem because of some underpinning issues. Given the phenomenon of incorporation we doubt whether even the most sophisticated requirements of informed consent can protect women from a camouflaged exploitation, because as Malmqvist and Zeiler rightly put it: “certain cultural norms conceal themselves and thus elude autonomous reflection” (Malmqvist and Zeiler 2010, p. 146). Also, Sonya Charles points out that decisions relied on “internalized oppressive norms” are not autonomous (Charles 210, p. 424).

Hence, it seems that it is very doubtful whether the structure of autonomous will is so ‘free of traps’ as Frankfurt seems to imply, saying that we are really free if we want what we want to want (Frankfurt 1971, p. 17). We claim that Frankfurt/Dworkin’s concept of the second order desires may not reflect people’s genuine preferences and wishes, but rather the ones determined by social and gender stereotypes. The background the theory of the second order desires has referred to seems to be falsified by some *idées reçues* of alleged women’s altruism, unselfishness, sympathy and commitment to the needs of others. It seems that the refusal of gamete donation for research stands in an utter opposition to practicing moral virtues embedded in that social and cultural image of a woman.

Thus, we think that even if the requirements matching the more demanding version of the concept of informed consent are adopted, it is still highly questionable whether woman’s decisions, even if they reflect her second order desires, are not clouded by the set of imprinted moral virtues. To approach the case slightly perversely, we can imagine a scenario where a woman’s first order desire is not to donate her eggs or spare embryo for research. Once she, however, reflects upon her first order desire, she finds out that she should want to want to donate them. In this scenario, paradoxically, it is rather ‘listening to her guts’ than referring to second order desires that properly reflects her genuine will.

The very essence of moral life is an inevitable tension between the quest for independence and the awareness of being entwined in a network of relations. Literature abounds with examples of feminine moral ideal that was so eloquently articulated by Woolf. Regardless of what we think of feminine and masculine moral ideals, this paper invites the readers to reflect on such ways of modeling feminine attributes that in a camouflaged way affect their decisions in the realm of ART. Given that countless generations of women have been brought up in accordance with the ideal of femininity described by Gilligan and criticized by Woolf, it seems obvious that in the case of women the language of gift and reference to altruism could be very persuasive.

In our opinion, only a genuine change of the dominant discourse will be able to overcome the detrimental results of these profound gender models that are in many cases absorbed unreflectively. To begin the process of individual and social changes the incorporated oppressive norms must become excorporated. As Malmqvist and Zeiler point out this process often commences with a sort of surprise when something treated so far as obvious is challenged (Malmqvis 2010, pp. 149–150). To end our paper with an optimistic view, we point to the fact that such a shift in the cultural narratives has been already unfolding in the mass-media. Maybe Merida the Brave who is the first Disney’s heroine to act individualistically, independently and egoistically can become the source of this elucidating surprise.
